# Ferroptosis and the eye: bridging the gap between cell death and vision preservation

**DOI:** 10.3389/fimmu.2026.1791087

**Published:** 2026-03-31

**Authors:** Guangyu Zhu, Yumeng Lin, Zhongyu Han, Haiyan Cao, Keyin Zhu, Ruijie Shi, Yuxuan Deng, Sizhen Li, Qingsong Yang, Xuejing Lu

**Affiliations:** 1 Eye School of Chengdu University of TCM, Chengdu, China; 2 Eye Health with Traditional Chinese Medicine Key Laboratory of Sichuan Province, Chengdu, China; 3 Retinal Image Technology and Chronic Vascular Disease Prevention and Control and Collaborative Innovation Center, Chengdu, China; 4 Department of Nanjing Tongren Eye Center, Nanjing Tongren Hospital, School of Medicine, Southeast University, Nanjing, China; 5 School of Medicine, Southeast University, Nanjing, China

**Keywords:** B cell, ferroptosis, immune modulation, macrophages, microglia, ocular microenvironment, T cells

## Abstract

Ferroptosis, a recently discovered type of programmed cell death (PCD) distinguished by iron overload and lipid peroxidation, differs fundamentally from necrosis, apoptosis, and autophagy. Emerging evidence indicates that ferroptosis is deeply implicated in the disruption of the ocular microenvironment, wherein both structural and immune cells are significantly compromised. Rather than being an isolated cellular event, ferroptosis actively engages in a complex bidirectional crosstalk with the ocular immune system, driving neuroinflammation and tissue degeneration. In this review, we shift the paradigm from traditional disease-specific descriptions to the underlying microenvironmental interactions that dictate ocular health. Furthermore, we systematically evaluate the therapeutic potential of emerging interventions. Specifically, we highlight the latest breakthroughs in utilizing natural and dietary compounds as potent ferroptosis regulators. Crucially, we address current translational limitations by exploring advanced drug delivery systems, such as nanocarriers and hydrogels, designed to effectively overcome the blood-retinal barrier (BRB) and improve targeted efficacy. Ultimately, this review provides a comprehensive roadmap for advancing ferroptosis-targeted therapies from the laboratory to clinical ophthalmology.

## 1 Introduction

With an aging global population, it is estimated that 2.2 billion people worldwide are suffering from visual dysfunction, which severely impedes the individual’s quality of life and poses threats to public health (1). The progressive degradation of certain cell types within the visual pathway, especially those such as retinal ganglion cells (RGCs) and retinal pigment epithelial (RPE) cells that are incapable of regeneration, is considered to be a primary driver of visual impairment. Regulated cell death (RCD), otherwise termed programmed cell death (PCD), is a form of cell death governed by specific biomacromolecules and could be modulated both genetically and pharmacologically (2). The RCDs implicated in ocular diseases comprise necrosis, apoptosis, autophagy, pyroptosis, and ferroptosis. The blockade or induction of these RCDs is considered to hold clinical potential for improving ocular cell viability and rendering ophthalmic therapy more efficient (3).

**Figure 1:**
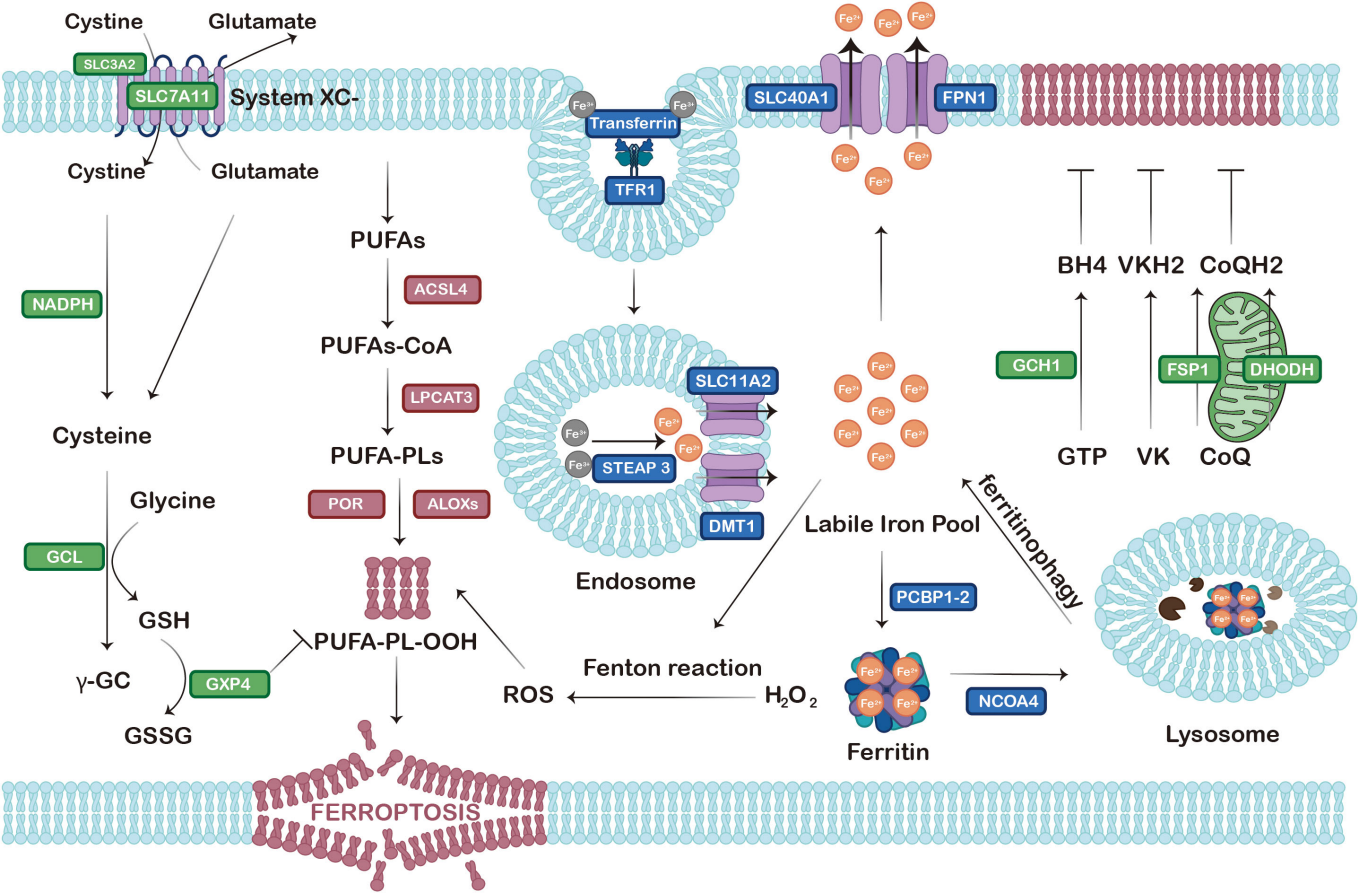
The core mechanisms of ferroptosis. Ferroptosis is characterized by three biochemical events: disturbances in iron metabolism, LPO, and antioxidant collapse. Core mechanisms include (1): disturbances in iron import (transferrin/TFR1, STEAP3/SLC11A2/DMT1), storage (PCBP1-2), and export (NCOA4/SLC40A1/FPN1) contribute to the overload of intracellular Fe²^+^, which serves as the initiating event in ferroptosis; (2) LPO mediated by enzymes (such as ACSL4, LPCAT3, and ALOXs) generates toxic PUFA-PL-OOH, which compromises the integrity of cell membranes, forming the core execution process of ferroptosis; (3) antioxidant defense systems include the system Xc-/GSH/GPX4 axis, the GCH1/BH4 axis, the DHODH/CoQH2 axis, and the FSP1/CoQH2 axis, the collapse of which fails to suppress LPO and ferroptosis. Proteins implicated in iron metabolism are labeled in blue; Proteins associated with LPO are labeled in red;Proteins involved in the antioxidant system are labeled in green. Abbreviations: SLC3A2, solute carrier family 3 member 2; SLC7A11, solute carrier family 7 member 11; SLC40A1, solute carrier family 40 member 1; FPN1, ferroportin 1; TFR1, transferrin receptor 1; PUFAs, polyunsaturated fatty acids; BH4, tetrahydrobiopterin; VKH2, hydroquinone vitamin K; CoQH2, ubiquinol; ACSL4, acyl-CoA synthetase long-chain family member 4; NADPH, nicotinamide adenine dinucleotide phosphate; PUFAs-CoA, polyunsaturated fatty acyl-CoA; GCH1, GTP cyclohydrolase 1; FSP1, ferroptosis suppressor protein 1; DHODH, dihydroorotate dehydrogenase; SLC11A2, solute carrier family 11 member 2; DMT1, divalent metal transporter 1; LPCAT3, lysophosphatidylcholine acyltransferase 3; PUFA-PLs, polyunsaturated fatty acid-containing phospholipids; GTP, guanosine triphosphate; VK, vitamin K; CoQ, coenzyme Q; STEAP3, six-transmembrane epithelial antigen of prostate 3; POR, cytochrome P450 oxidoreductase; ALOXs, lipoxygenases; LIP, labile iron pool; GCL, γ-glutamylcysteine ligase; PCBP1-2, poly (rC)-binding proteins 1 and 2; GSH, glutathione; PUFA-PL-OOH, polyunsaturated fatty acid-containing phospholipid hydroperoxides; H_2_O_2_, hydrogen peroxide; γ-GC, γ-glutamyl cysteine; GPX4, glutathione peroxidase 4; ROS, reactive oxygen species; NCOA4, nuclear receptor coactivator 4; GSSG, glutathione disulfide.

Ferroptosis, a term initially coined by Dixon in 2012, is a distinctive form of RCD featured by the iron-dependent lethal aggregation of lipid peroxides located at the cell membrane (4) (**Figure 1**). In contradistinction to autophagy and apoptosis, cells subjected to ferroptosis manifest specific cytological alterations, namely dysmorphic mitochondria with condensed membrane, diminished/vanished cristae, and ruptured outer membrane (5, 6). The abnormalities in cell functions are attributed to the peroxidated membrane phospholipids (PLs) containing polyunsaturated fatty acids (PUFA-PLs), which have been shown to result in a compromised selective permeability of the plasma membrane (7). Identified as a node connecting cell physiology and redox biochemistry, ferroptosis was implicated in a diverse array of pathophysiological processes involving ischemic organ injury, neurodegenerative diseases, and carcinogenesis (8).

In the event of ocular diseases, RCD has been observed to be predominantly executed through apoptosis. Nevertheless, emerging evidence indicates that ferroptosis, despite its absence of the biochemical and genetic features of apoptosis, contributes to the deterioration of RPE cells, RGCs, photoreceptor (PR) cells, corneal epithelial cells (CECs), etc. For instance, Qin and colleagues assessed the impact of necroptosis, apoptosis, and ferroptosis inhibitors on RGCs subjected to retinal ischemia reperfusion (RIR) injury, with Ferrostatin-1 (Fer-1) conferring the superior efficacy (9). In parallel, an intricate interaction occurs between ferroptosis and immune cells within the eye has been unveiled. The damage-associated molecular patterns (DAMPs) released by ferroptotic cells could activate and recruit macrophages, alerting immune defenses (10). Additionally, the occurrence of ferroptosis may facilitate immune evasion by driving microglia polarization and triggering the ferroptosis of CD8+ T cells (11).

Despite the growing body of literature on this topic, a significant translational gap remains. While our previous work (12) outlined the fundamental molecular pathways of ferroptosis in eye diseases, this review advances the field by shifting the focus toward the complex bidirectional crosstalk between ferroptosis and the ocular immune microenvironment. We emphasize how ferroptotic cells and resident immune populations, particularly microglia and other glial cells, interact to either exacerbate inflammatory damage or promote tissue repair.

Furthermore, to bridge the gap between basic research and clinical application, we uniquely highlight the latest breakthroughs in natural/dietary compounds and advanced drug delivery systems (e.g., nanocarriers and hydrogels) designed to overcome the blood-retinal barrier. Traditional systemic administration of anti-ferroptotic agents often faces poor ocular bioavailability and non-targeted toxicity. By systematically addressing these microenvironmental dynamics and physical translational hurdles, this review aims to provide a refined conceptual framework and practical engineering solutions for the next generation of ferroptosis-targeted ocular therapies.

## 2 Ferroptosis and immune modulation

Ferroptosis and immune modulation (both innate immunity and adaptive immunity) engage in bidirectional crosstalk. Innate immunity is the body’s first defense against invading pathogens and comprises several types of cells, such as macrophages, neutrophils, and dendritic cells. Ruptured ferroptotic cells trigger innate immunity by releasing damage-associated molecular pattern molecules (DAMPs), such as heat shock proteins (HSPs) and oxidized lipids, upon tissue damage or tumor therapy (13). This recognition promotes the secretion of pro-inflammatory cytokines and enhances the body’s resistance to infection by pathogens and tumor progression (14). Excessive release of DAMPs, however, induces overactivation of innate immune cells, forming an inflammatory storm and exacerbating tissue damage (13). In turn, activation of macrophages leads to the secretion of interferon-γ (IFN-γ), which can downregulate the expression of SLC7A11 and inhibit GSH synthesis, thereby promoting ferroptosis and clearing infected and tumor cells (15). Additionally, dendritic cells can indirectly modulate the progression of ferroptosis by regulating the transcription of genes related to iron metabolism (e.g., TF and FPN1) during antigen presentation (16).

Composed of B cells, T cells, and their secreted products, adaptive immunity is responsible for recognizing and clearing pathogens or abnormal cells specifically and forming immune memory (17). GPX4 functions as a critical mediator between ferroptosis and adaptive immune cells. It facilitates the maturation, survival, and antibody response of B cells in the peritoneum, pleura, and marginal zone, with the ablation of it inducing ferroptosis and weakening the IgM antibody response (18, 19). Moreover, evidence suggests that the overexpression of GPX4 or the depletion of ACSL4 confers protection to CD8+ T cells against ferroptosis (20). However, ferroptotic cancer cell-secreted GPX4 could bind to zona pellucida glycoprotein 3 (ZP3) on the surface of dendritic cells, and instead suppress anti-tumor T cell activation (21). Additionally, ferroptosis-induced lipid peroxides can determine the differentiation and activity of CD4+ T cells into pro-inflammatory or anti-inflammatory subsets, thereby shaping the direction of adaptive immune responses (22).

### 2.1 Macrophages

**Figure 2:**
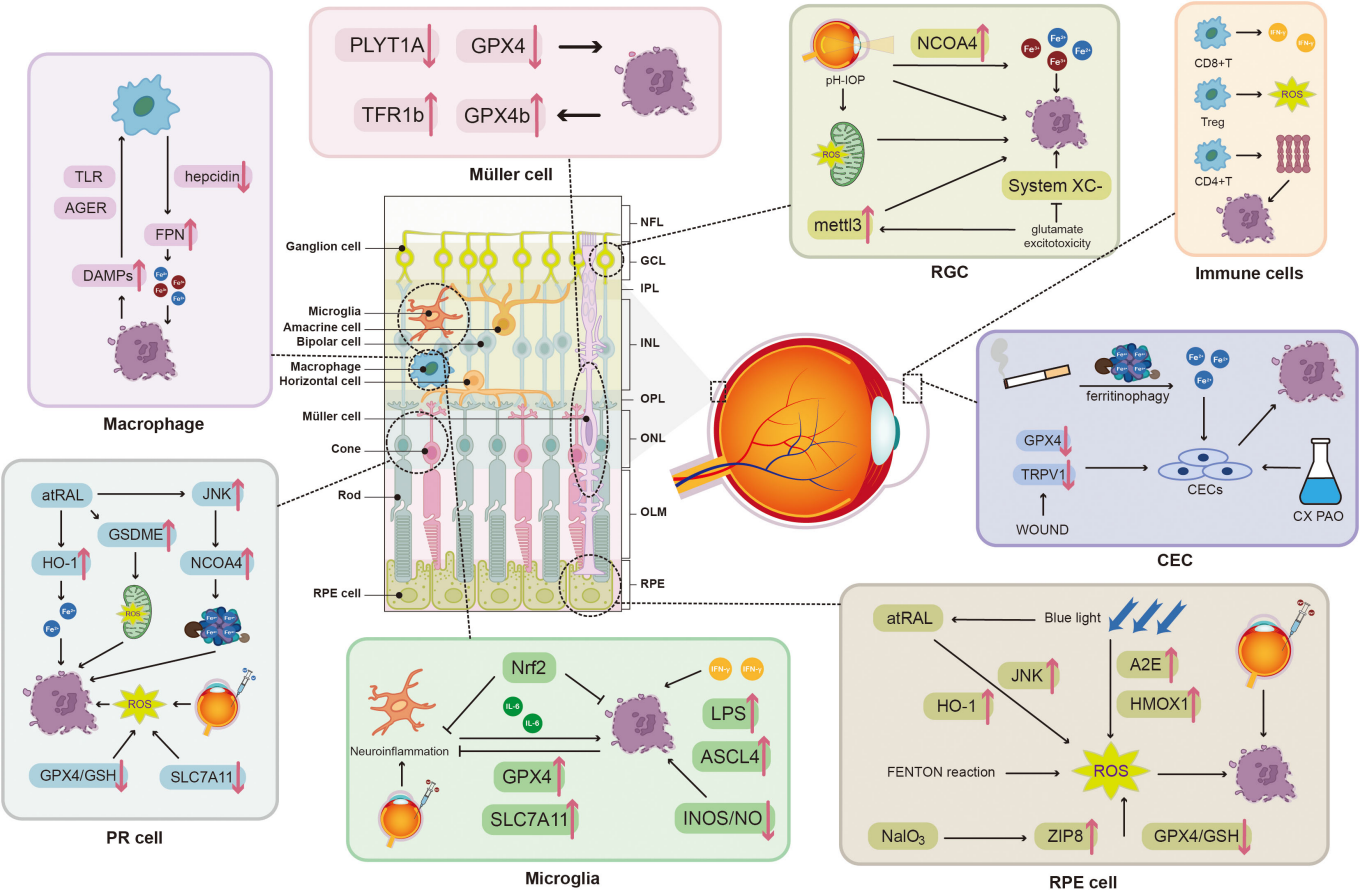
Ferroptosis in the ocular microenvironment. Ferroptosis, induced by distinct mechanisms, interacts reciprocally with both structural cells (RPE cells, RGCs, PR cells, and CECs) and immune cells (macrophages, microglia, and T cells) within the ocular microenvironment: (1) ferroptosis disrupts the integrity and the function of structural cells, thereby compromising visual acuity; (2) ferroptotic cells release DAMPs that shape the activation and polarization of immune cells, which in turn secrete cytokines that modulate the molecular regulation of ferroptosis. Abbreviations: ph-IOP, Pathologically high intraocular pressure; TLR, toll-like receptor; AGER, advanced glycosylation end product-specific receptor; mettl3, methyltransferase-like 3; NFL, nerve fiber layer; GCL, ganglion cell layer; DAMPs, damage-associated molecular patterns; IPL, inner plexiform layer; INL, inner nuclear layer; OPL, outer plexiform layer; ONL, outer nuclear layer; TRPV1, transient receptor potential vanilloid 1; atRAL, all-trans-retinal; OLM, outer limiting membrane; GSDME, gasdermin E; CX, phosgene oxime; PAO, phenylarsine oxide; HO-1, heme oxygenase-1; A2E, N-retinylidene-N-retinylethanolamine; Nrf2, nuclear factor erythroid 2-related factor 2; HMOX1, heme oxygenase 1; LPS, lipopolysaccharide; IL-6, interleukin-6; INOS, inducible nitric oxide synthase; NO, nitric oxide; NalO3, sodium iodate; ZIP8, zinc transporter 8.

Macrophages, which form a pivotal element of the innate immune system, are imperative for several facets of inflammatory processes, phagocytosis of debris, and tissue repair (23). Depending on their provenance, macrophages in the retina are categorized into two primary groups: intrinsic macrophages (situated within the vitreoretinal interface and the retina) and recruited macrophages (deriving from infiltrating monocytes) (24) (**Figure 2**).

It has been hypothesized by certain researchers that there is an interaction between retinal macrophages and ferroptosis, given the resemblance in the functions of the macrophages and the traits of ferroptosis (25). To begin with, certain cytokines secreted by macrophages have been demonstrated to induce or inhibit ferroptosis by controlling the transcription of hepcidin and modulating the expression of ferroportin (FPN). Excessive hepcidin causes increased binding to FPN, the only identified mammalian intracellular iron exporter, leading to increased internalization and degradation of hepcidin-FPN that aggravates iron deposition in cells (26). Within the retina, an increase in hepcidin of endothelial cells (ECs) prepared from Cyp1b1-/- mice was found to lead to a reduction in the levels of FPN (27). As indicated in a separate article, following the injection of intraperitoneal iron dextran, the retina-specific hepcidin KO induced elevated levels of iron accumulation in retinal vascular ECs and RPE cells, but not in neurosensory retinal cells (28). It has been reported that either the knockdown or pharmacological intervention of IL-6, a symbolic inflammatory marker secreted by M1 macrophages, curtailed the binding of hepcidin with FPN, attenuating the ubiquitination of FPN (29). Another marker of pro-inflammatory macrophages, namely IL-1β, has previously been shown to upregulate the levels of FPN in glial cells, thereby promoting iron deposits in neurons and the surrounding environment (30).

Macrophages’ phagocytosis contributes considerably to the removal of ferroptotic cells, which could release a DAMP called HMGB1 to activate and recruit macrophages, alerting immune defenses (10). The overexpression of HMGB1, a primary DAMP that contributes to aseptic inflammation, increased the expression of markers of M1-phenotype macrophages (CD16 and iNOS) as well as reducing the levels of markers of M2-phenotype macrophages (Arg-1 and CD206) in the retina (31). Moreover, the neuroinflammatory injury of RGCs caused by macrophages’ activation after ONC was alleviated by intravitreal injection of the HMGB1 inhibitor BoxA (32). Conversely, conditional KO of HMGB1 in rods restored the thickness of the ONL, yet had no effect on the mobilization and activation of macrophages typically seen following retinal detachment, suggesting the potential involvement of other DAMPs in the process (33).

In the context of ferroptosis, either advanced glycosylation end-product specific receptor (AGER) or Toll-like receptors (TLR) on macrophages is required for DAMPs to mediate inflammation. The secretion of TNF-α and IL-1β induced by HMGB1 in PR cells was blocked by FPS-ZM1 (an inhibitor of the AGER), thus reversing the degeneration of PR in rd1 mice (34). Oxidized PLs (another type of DAMPs) on the membrane of ferroptotic cells could interact with TLR2 on macrophages, enhancing the phagocytic efficiency of the cells (10). In addition to ferroptotic cells, the overloaded iron produced during ferroptosis could also determine the development and differentiation of macrophages. As with DAMPs, iron accumulation in macrophages, the cells responsible for engulfing red blood cells (RBCs) and then storing iron by binding it to ferritin, is prone to provoking M1 polarization through the induction of inflammatory factors, enhanced glycolysis, p53 acetylation, etc. (10).

### 2.2 Microglia

As the specialized resident macrophages in the neural retina, microglia constitute a primary population of glial cells (35). Among diverse cell types within the CNS, microglia are distinguished by their remarkable capacity for iron storage and iron accumulation under pathological conditions (36). In a recently conducted study that explored the vulnerabilities of neurocytes to ferroptosis, Jiao and his colleagues revealed that microglia were the most sensitive to ferroptosis (37).

**Figure 3:**
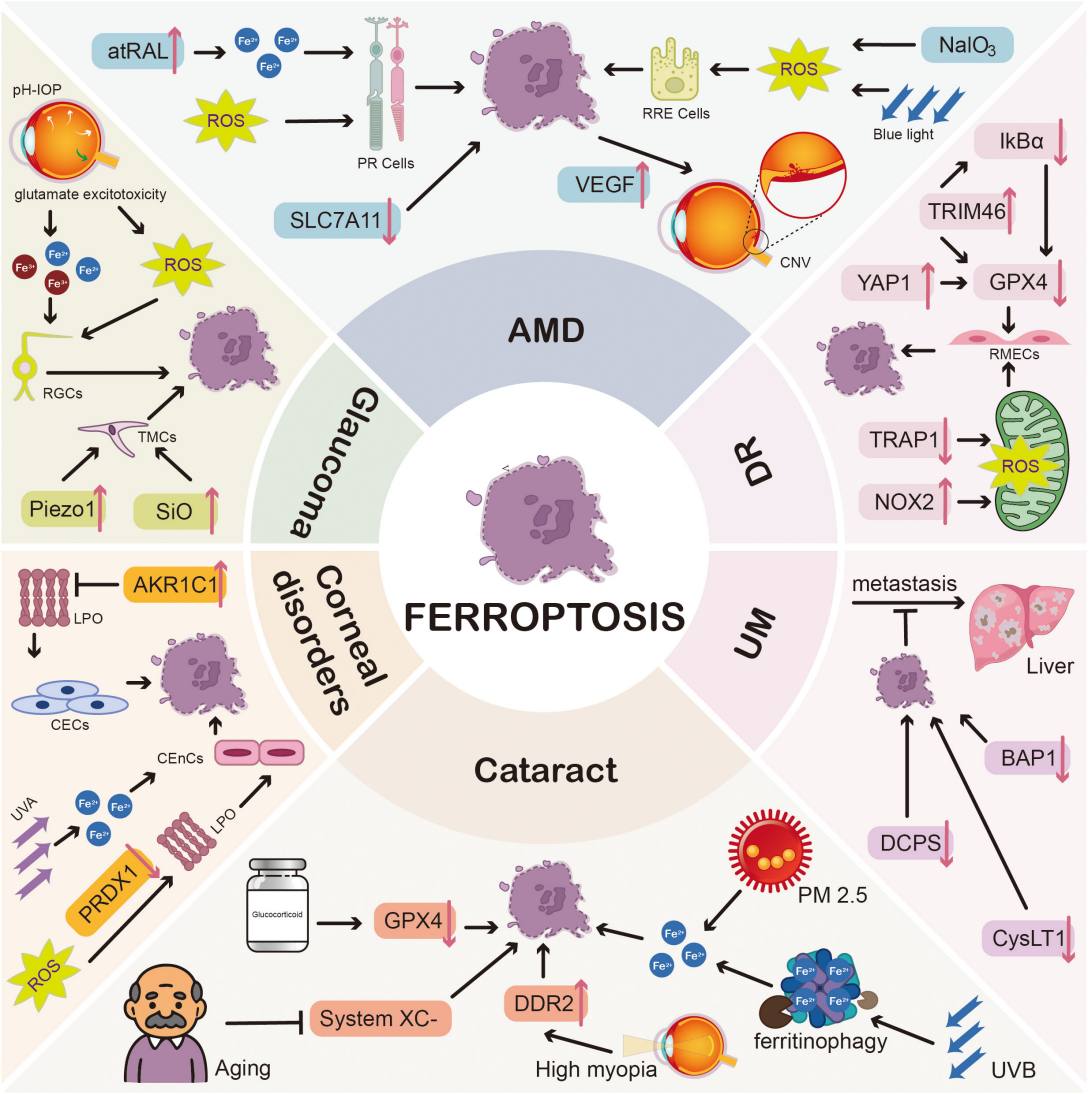
Ferroptosis in ocular diseases. A variety of risk factors and protein/gene alterations affect cells within the ocular microenvironment via specific molecular mechanisms, eventually triggering ferroptosis and the onset of six ocular diseases (DR, AMD, glaucoma, cataract, corneal disorders, and UM). For example, in glaucoma: (1) pH-IOP or glutamate excitotoxicity stimulates the release of Fe²^+^, Fe³^+^, and ROS, inducing ferroptosis of RGCs and impairing visual acuity; (2) the upregulated levels of Piezo1 or SiO trigger ferroptosis of TMCs, resulting in higher IOP.Abbreviations: IkBα, inhibitor of nuclear factor kappa-B alpha; TRIM46, tripartite motif-containing 46; VEGF, vascular endothelial growth factor; CNV, choroidal neovascularization; YAP1, yes-associated protein 1; TRAP1, tumor necrosis factor receptor-associated protein 1; NOX2, NADPH oxidase 2; Piezo1, piezo type mechanosensitive ion channel component 1; SiO, silicone oil; AKR1C1, aldo-keto reductase family 1 member C1; BAP1, BRCA1-associated protein 1; DCPS, decaprenyl diphosphate synthase subunit; PM2.5, particulate matter 2.5; CysLT1, cysteinyl leukotriene receptor 1; DDR2, discoidin domain receptor 2; UVB, ultraviolet B.

Despite the absence of consensus on the mechanisms underlying microglial ferroptosis, several genes implicated in this process have been identified. The gene silencing of ACSL4 in primary microglial cells has been reported to impede ferroptosis of microglia induced by HIV-1 Tat protein, with an effect comparable to that of the administration of Fer-1 or DFO (38). In the cell model of AMD, the knockdown of the ferroptosis-related gene (FRG) ANXA1, whose colocalization with IBA1 was observed by immunofluorescence assays in mouse tissue, facilitated the reduction of GPX4 (39) (**Figure 3**).

Apart from the genetic susceptibility to ferroptosis, microglial sensitivity to ferroptosis also depends on its state and was affected by iNOS/NO·. Under pro-inflammatory conditions, as evidenced by interventions with LPS or IFN-γ, microglia are insensitive to ferroptosis, yet susceptible to iron overload (40). It has been shown that microglia exposed to LPS displayed a primed phenotype with heightened levels of inflammatory markers such as iNOS, IL-1β, and p65, which were accompanied by iron deposits and ferroptosis (41). In an experimental uveitis model, LPS stimulation was observed to induce the upregulation of 14-3-3zeta and the stabilization of HO-1, thus prompting microglial ferroptosis in retinal inflammation (42). Furthermore, the *in vitro* data showed that genetic depletion of iNOS renders Raw264.7 cells and EOC 20 cells vulnerable to ferroptosis, whereas NO· donors (SNAP, DPTA-NONOate) empower the ferroptotic resistance of M2 microglia (43). In the retinal degeneration model of light-treated BALB/cJ mice, treatment with indole-3-carbinol diminished INOS and NO· released by microglia, while concomitantly increasing the levels of anti-oxidants gene NQO1 and HMOX1.

The sophisticated interplay between neuroinflammation, instigated by microglia in response to inflammatory stimuli, and ferroptosis in microglia has been documented. In BV2 cells, the inflammatory factor IL-6 was upregulated after FAC exposure, in turn, DMT1 was upregulated and FPN1 was downregulated following 24 hours of IL-6 stimulation, which resulted in iron overload (44). In a study employing OGD/R models of BV2 cells, the intervention of vitamin K1 attenuated retinal inflammatory responses by moderating microglial ferroptosis, which was characterized by increased level of GPX4 and SLC7A11 (45). The inhibition of Nrf2, an antioxidant transcription factor, has been evidenced to ameliorate ferroptosis in microglia induced by PM2.5 exposure. Research has also demonstrated that the Nrf2 inhibitor could eliminate PM2.5-induced neuroinflammation as well as the upregulation of the gene HO-1, suggestive of Nrf2 as a mediator of inflammation and microglial ferroptosis (46).

Oxidative stress and autophagy were reported to participate in microglial ferroptosis likewise. The knockdown of microglial S100A8, a protein mediating cellular damage by enhancing oxidative stress and autophagy, was shown to inhibit the expression levels of ROS, MDA, and ferroptotic indicators, namely ACSL4, NOX2, and 15-LOX (47).

### 2.3 T cells

The delicate equilibrium between the activation and elimination of T cells is finely tuned to ensure efficacious immune responses and avert autoimmune diseases. Extensive evidence has confirmed that specific ferroptosis-related metabolic signals or byproducts were involved in the function, viability, and differentiation of multiple T-cell subgroups, impacting their reactivity to inflammation, infection and tumor immunity (48).

As was reported recently in tumor immunity, the ferroptotic interaction between CD8+ T cell-secreted IFN-γ and tumor cells is known to induce the elicitation of tumor ferroptosis and reinforcement of anti-tumor activity (49). A nanopore long-read sequencing approach was employed to elucidate the anti-tumor effects of UM-specific neoepitopes, which were regarded as prospective targets in the immunotherapy of cancer. The findings revealed that these neoepitopes exerted immune responses against this ocular malignancy by stimulating the production of IFN-γ and triggering T cell-based tumor killing (50). Subsequent to the photodynamic therapy on vivo models of UM’s pigmentation, the presence of enhanced IFN-γ positive CD8+ T cells, accompanied by increased exposure of ferroptosis-related DAMPs, were detected in both the pigmented tumors and the draining lymph nodes (51).

Besides, regulatory T (Treg) cells with a deletion of PDK1 have been implicated in the generation of excessive ROS, which consequently leads to amplified ferroptosis of themselves (52). On the other hand, the restoration of redox homeostasis could likewise modulate the imbalance of T helper 17 (Th17)/Treg cells, thereby ameliorating experimental autoimmune uveitis (EAU) (53). *In vitro* study corroborated that the suppression of the ROS/TXNIP/HIF-1α pathway, which was presumably associated with the mitochondrial dysfunction in ferroptosis, fosters the ratio of retina-specific Treg cells, notably attenuating ocular inflammation and optic disc edema (54).

Certain regulators of ferroptosis have emerged as vital contributors in the disruption of the proliferation and survival of CD4+ T cells that potentially exacerbate both tumor progression and pathogen invasion (55). Analysis of the lipid deposition patterns in immune cells revealed that, compared with myeloid cells, CD4+ T cells were more enriched in PUFA-PLs (substrates of LPO), making these cells highly sensitive to ferroptosis (56). It was established that carboxyethylpyrrole (a LPO product) induces retinal infiltration of macrophages, a pathological process that is blocked by the immunosuppressive therapy of CD4+ T cells (specifically Th1 and Th17 cells) (57). Interestingly, the infiltration of Th1 and Th17 cells into the CNS, as well as the secretion of cytokines by CD4+ T cells, could also be initiated by ferroptotic neurons, which were induced by over-expression of ACSL4 (a regulator of PUFA-PLs synthesis) (58). In mouse models of oxygen-induced retinopathy (OIR), low-dose IL-2, which has been shown to upregulate surface TFR1 expression, was observed to expand CD4+CD25+Foxp3+ Tregs, thereby effectively attenuating retinal vasculopathy (59, 60). Moreover, TFR1 blocking monoclonal antibody, the administration of which hinders iron endocytosis and triggers combined immunodeficiency, was reported to activate altruistic and non-proliferating T cells (61). As previously illustrated, the production of mt-ROS is the key process in ferroptosis-induced RGC death, which could be reversed by the use of the mitochondria-selective antioxidant MitoTEMPO (62). Mechanistic studies revealed that the attenuation of ROS from CD4+ T cells resulted in a reduction in EAU, which was achieved by constraining the polarization of T cells towards Th17 and Th1 cells within the retina (54).

### 2.4 Other immune cells

The interplay between other immune cells and ferroptosis in ocular immune microenvironment remains to be thoroughly explored, although some bioinformatics analyses have commenced in these domains. Two bioinformatics analyses identified three (ACO1, HCRA1, and MMD) and six (CYBB, CTSB, SLC38A1, TLR4, PEX2, and ABCC1) FRGs, respectively, with strong diagnostic specificity in the orbital fibroblasts of thyroid-associated orbitopathy (TAO) patients (63, 64). The assessment of correlation between these genes and infiltrating immune cells demonstrates that ACO1 and MMD are inversely associated with infiltration of activated mast cells, while ABCC1, CYBB and PEX2 manifest a substantial positive relation with resting mast cells. An investigation revealed that specific tumor-infiltrating immune cells (primarily resting mast cells) displayed a negative correlation with a FRG signature, which has the capacity to precisely predict the prognosis of UM (65). Furthermore, ferroptosis induced by mast cells was identified as a driving force in the progression of Fusarium keratitis, with the hub genes HMOX1, CYBB, and ALOX5 correlating markedly with these immune cells in the spearman analysis (66).

In the context of B cells, NOX4 and PARP14, both of which were identified as common ferroptosis differentially expressed genes, were associated with immune infiltration of activated B cells in DR patients (67). A CIBERSORT analysis revealed that the proportions of memory B cells and resting dendritic cells in orbital adipose tissue of TAO patients were markedly elevated, correlating positively with the optimal feature genes of ferroptosis in TAO (63). In keratoconus, CD56 natural killer cells were observed to be regulated by AKR1C3, the depletion of which might result in a reduction of SLC7A11 expression and subsequent interruption of the system Xc- (68).

Owing to the presence of BRB and the absence of lymphatics maintaining the immune privilege of the retina, various glial cells (Müller cells, astrocytes, etc.), despite not being classified as immune cells, contribute extensively to the ocular immunity. Disruption of iron chelation has been reported to cause influx of proliferating Müller cells in the zebrafish retina, a process requiring both TFR1b and the GPX4b (69). Proteomic and bioinformatic analyses illustrate that deficiency in the PCYT1A, a gene abundantly expressed in Müller cells of the INL, dysregulates the activity of the metabolism of retinal fatty acids and ferroptosis-related pathways (70). Zeng et al. identified a particular subset of Müller cells rich in Sox2, the number of which decreased by ferroptosis and apoptosis as AMD progressed (71). Furthermore, the ferroptosis of Müller cells, manifested by reduced GPx4 and increased intracellular Fe^2+^, was considered to be the cause of complications related to vitrectomy with silicone oil (SiO) endotamponade (72). A research group has investigated the mechanism underlying ferroptosis of Müller cells under conditions of high-glucose (HG). The findings suggested that the axis of AQP4/TRPV4 and SQSTM1/ACSL4 were implicated in the ferroptosis of Müller cells in the context of DR (73, 74). In the initial stages of DR, the anti-ferroptotic effects of resveratrol on Müller cells via the Nrf2/GPx4/PTGS2 pathway have been elucidated (75). Ferroptosis in astrocytes or oligodendrocytes has also been proven to mediate multiple neurodegenerative diseases, including spinal cord injury, Parkinson’s disease, and ischemic stroke (76–78).

## 3 Ferroptosis and non-immune modulation

### 3.1 RPE cells

Serving as a monolayer of pigmented cells between the neurosensory retina and the choriocapillaris, RPE maintains subretinal homeostasis by performing multifaceted functions, including forming the blood-retinal barrier (BRB), transporting nutrients and growth factors, and phagocytosing PR outer segments (79). The progressive degeneration or dysfunction of RPE cells, which is a hallmark of multiple retinal disorders, invariably causes vision loss or even permanent blindness due to the absence of regenerative capacity (80) (**Table 1**).

**Table 1:** Cell-specific ferroptosis in ocular diseases.

Ocular diseases	Affected cell types	Ferroptotic mechanism	Outcome	References
AMD	RPE cells	The exposure of A2E to blue light elevates Fe²^+^ levels and inhibits the SLC7A11-GSH-GPX4 axis.	Ferroptosis of RPE cells	(87)
	RPE cells	Excessive superoxide activates the Haber-Weiss reaction, triggering the Hsp70-linked ubiquitin-dependent degradation of GPX4.	Ferroptosis of RPE cells	(88)
	PR cells	The overload of atRAL increases ACSL4 levels and inhibits System Xc^-^.	Ferroptosis and atrophy of PR cells	(107)
	PR cells and RPE cells	The overload of atRAL elevates Fe²^+^ levels and facilitates ROS overproduction by activating HO-1.	Ferroptosis of PR cells; Atrophy of RPE cells	(108)
	PR cells	The overload of atRAL induces ferritinophagy by activating JNK signaling and promoting the expression of NCOA4.	Ferroptosis of PR cells; Degeneration of the retina	(109)
	PR cells	The overload of atRAL triggers mitochondrial ROS production, iron overload, and LPO by activating GSDME.	Ferroptosis of PR cells; Degeneration of the retina	(110)
	PR cells and RPE cells	QDs inhibit the expression of PRPF8, leading to the unsplicing of GPX4b and the decrease of GSH.	Ferroptosis of PR cells and RPE cells; Differentiation of PR cells	(111)
	PR cells	The suppression of the SLC7A11-GPX4 pathway leads to iron accumulation, cystine depletion, and enhanced LPO.	Ferroptosis of PR cells; Impairment of visual function	(113)
	RPE cells	The upregulation of IFN-γ increases intracellular Fe²^+^ by blocking System Xc^-^ via the activation of the JAK1-2/STAT1/SLC7A11 pathway.	Ferroptosis of RPE cells	(151)
	RPE cells	The elevation of mtDNA copy numbers induces ferritinophagy and increases the PUFA levels.	Improvement in pro-ferroptotic activity of RPE cells	(166)
	RPE cells	The knockdown of NRF2 decreases the expression of SLC7A11, increasing the level of LPO.	Ferroptosis of RPE cells; Expansion of the CNV area	(167)
DR	RPE cells	Abnormalities in the autophagy-lysosome degradation of ACSL4 produce lethal lipid species.	Ferroptosis of RPE cells; Impairment of the visual function	(91)
	Müller cells	The upregulation of AQP4 positively regulates TRPV4 expression, which enhances ferroptosis.	Ferroptosis of Müller cells; Injury of the retina	(73)
	Müller cells	The elevated levels of SQSTM1 exacerbate ferroptosis via ACSL4 upregulation.	Ferroptosis and mitochondrial damage of Müller cells	(74)
	RMECs	HG treatment leads to higher levels of FTH1, TFR1, ACSL4, GPX4, and ROS.	Ferroptosis of RMECs; Dysfunction of the retinal microvasculature	(168)
	RMECs	The overexpression of TRIM46 promotes GPX4 ubiquitination.	Ferroptosis of RMECs; Dysfunction of the retinal microvasculature	(169)
	RMECs	HG treatment facilitates the elevation of ACSL4, TFRC and the decline in GPX4.	Ferroptosis and inflammation of RMECs	(170)
	RMECs	The downregulation of PCBP1 leads to an imbalance in iron homeostasis as well as an aberrant level of iron regulatory proteins by activating the HIF-1α/HO-1 pathway.	Ferroptosis of RMECs	(171)
	RMECs	The suppression of FLOT1 stimulates the Nrf2 pathway, which activates the SLC7A11/GPX4 axis to inhibit LPO.	Ferroptosis of RMECs; Damage of BRB	(172)
	RMECs	The knockdown of NOX2 initiates the damage of mitochondria, inducing the self-perpetuating vicious cycle of ROS.	Mitochondrial ferroptosis of RMECs	(173)
	RPE cells	The reduction in TRAP1 expression aggravates the mitochondrial damage by inhibiting the interactions with ACSL1, ACSL4, and CYB5R1.	Mitochondrial ferroptosis of RPE cells	(174)
Glaucoma	RGCs	ph-IOP leads to abnormal accumulation of Fe²^+^ by the degradation of FTH1 mediated by NCOA4.	Ferroptosis of RGCs; Impairment of visual function	(95)
	RGCs	The downregulation of GPX4, accompanied by increased ROS and lipid peroxides, occurs in the mitochondria.	Ferroptosis of RGCs; Dysfunction of the retina	(62)
	RGCs	The M6A modification of HMGCS1 mediated by METTL3 promotes glutamate excitotoxicity-induced ferroptosis.	Ferroptosis of RGCs; Impairment of visual function	(101)
	RGCs	The expression of SAT1 and p-p38 MAPK/p38-MAPK is increased and levels of GPX4, SLC7A11, and ferritin light chain are decreased.	Ferroptosis of RGCs; Impairment of visual function	(102)
	RGCs	The deficiency in TF facilitates FeSO_4_-induced toxicity and NMDA-induced excitotoxicity.	Ferroptosis of RGCs; Injury of RGCs’ axons	(175)
	RGCs	The deletion of GLAST and excitotoxicity induced by glutamate promotes the enrichment of ferroptosis-related pathways.	Ferroptosis of RGCs; Abnormalities in retinal structure, thickness, and electrophysiology	(176)
	RGCs	GGT1 knockdown exacerbates autophagy by the disruption of the interaction with GCLC, leading to ferroptosis.	Ferroptosis of RGCs	(177)
	TMCs	Yoda1 induces ferroptosis, which relies on Piezo1 activation via the JNK/p38 axis.	Ferroptosis of TMCs	(178)
	TMCs	The ROS/NOX4/Smad3 axis mediates SiO-induced ferroptosis and the production of ROS.	Ferroptosis of TMCs; Fibrosis of the trabecular meshwork	(179)
Cataract	LECs	The elevated concentrations of PM2.5 modify the expression of ferroptosis-related genes.	Ferroptosis of LECs; Morphological and functional disorders of the lens	(180)
	LECs	Both SLC7A11 and SLC3A2 (cystine/glutamate antiporter subunits) and SLC40A1 (iron exporter ferroportin) are downregulated.	High susceptibility of LECs to ferroptosis	(181)
	LECs	The deficiency in glycine results in the over-degradation of ferritin and the obstruction of PCBP2-mediated Fe²^+^ transportation.	Ferroptosis of LECs	(182)
	LECs	The dysregulation of Notch signaling compromises the Nrf2/GPX4 antioxidant axis.	Ferroptosis of LECs	(183)
	LECs	circTFRC-236aa coded by circTFRC drives ferroptosis via the activation of the p62/Keap1/Nrf2 axis.	Ferroptosis of LECs	(184)
	LECs	The M6A-modified lncRNA regulates the expression of GPX4 by a cis mechanism.	Ferroptosis of LECs	(185)
Corneal disorders	CEnCs	The loss of expression in NRF2 and PRDX1 causes LPO.	Ferroptosis of CEnCs	(186)
	CEnCs	The upregulation of PTEN inhibits ferroptosis via the PI3K/Akt/mTOR axis.	Ferroptosis of CEnCs	(187)
	CEnCs	The siRNA knockdown of ferredoxin 1 increases susceptibility to ferroptosis, which is mediated by ISC and IREBP.	High susceptibility of CEnCs to ferroptosis	(188)
	CECs	The reactivation of DDR1 increases the levels of LPO and ACSL4 via the Hippo-YAP pathway.	Ferroptosis of CECs; Exacerbation of dry eye symptoms	(189)
	CECs	The downregulation of AKR1C1 by blocking NRF2 increases LPO and ferroptosis-induced cell damage.	Ferroptosis of CECs; Defects of the cornea	(190)
UM	UM cells	The inhibition of DCPS induces ferroptosis through the reduction of GLRX mRNA turnover.	Ferroptosis of UM cells; Elimination of stem-like cells in UM	(191)
	UM cells	The upregulation of CysLT1 promotes key markers that induce ferroptosis.	Ferroptosis of UM cells	(192)

Hsp70, Heat shock protein 70; JNK, c-Jun N-terminal kinase; IFN-γ, Interferon-γ; JAK1-2, Janus kinase 1 and 2; STAT1, Signal transducer and activator of transcription 1; AQP4, Aquaporin 4; TRPV4, Transient receptor potential vanilloid 4; SQSTM1, Sequestosome 1; FTH1, Ferritin heavy chain 1; HIF-1α, Hypoxia-inducible factor 1α; FLOT1, Flotillin 1; CYB5R1, Cytochrome b5 reductase 1; M6A, N^6^-Methyladenosine; HMGCS1, 3-Hydroxy-3-methylglutaryl-CoA synthase 1; SAT1, Spermidine/spermine N¹-acetyltransferase 1; p38 MAPK, p38 mitogen-activated protein kinase; NMDA, N-Methyl-D-aspartate; GLAST, Glutamate aspartate transporter; GGT1, γ-Glutamyltransferase 1; NOX4, NADPH oxidase 4; Smad3, Mothers against decapentaplegic homolog 3; PTEN, Phosphatase and tensin homolog; PI3K, Phosphatidylinositol 3-kinase; Akt, Protein kinase B; mTOR, Mammalian target of rapamycin; ISC, Iron-sulfur cluster; IREBP, Iron-responsive element-binding protein; PRDX1, Peroxiredoxin 1; DDR1, Discoidin domain receptor 1; YAP, Yes-associated protein; GLRX, Glutaredoxin; QDs, Quantum dots; PRPF8, Pre-mRNA processing factor 8.

Although the cell death mechanism of RPE, which is susceptible to oxidative stress, remains controversial, ferroptosis has been recently recognized as a primary mode of RPE cell death (81). The detached PR outer segments, 10% of which is phagocytosed daily by RPE, and the membrane of the RPE cells and are both enriched with PUFAs, the main substrates for LPO (82). Along with the RPE cells’ high metabolic demand mentioned previously, which depends on free iron as an imperative cofactor and produces considerable amounts of ROS, these findings firmly correlate ferroptosis with RPE cell death. In C57BL/6J mice given intravitreal injection of ferric ammonium citrate (FAC), which prompted the accumulation of LPO carboxyethyl pyrrole in RPE cells, these cells died in patches and then expanded at the borders (83). Moreover, a preceding absence of cell polarity was found in GPx4fl/fl mice subjected to an RPE-specific adeno-associated virus (AAV)-Cre vector, which was alleviated by the Fer-1 (84).

Sodium iodate (NaIO_3_), a common oxidative stressor that induced the generation of intracellular ROS, has been extensively employed to model selective RPE cell damage. Liu et al. investigated the traits of cultured human retinal pigment epithelium (ARPE-19) cells exposed to NaIO_3_ and observed that the process of cell death was concomitant with the deficiency of reduced thiol groups (particularly GSH and cysteine) as well as the amplified release of labile iron, which replicated the critical features of ferroptosis (85). Another report suggested that FSP1/CoQ10/NADPH and GSH/GPX4 pathways were compromised in RPE cells treated with NaIO_3_, which was exacerbated by silencing FSP1 and GPX4 (85). Pigment epithelium-derived factor (PEDF), the overexpression of which upregulated GPX4 and FTH1 that proved to suppress LPO and ferroptosis induced by NaIO_3_, was recently viewed as an upstream regulatory molecule of RPE ferroptosis (86). Collectively, these evidence supports the hypothesis that NaIO_3_-induced ROS caused the damage to RPE cells by ferroptosis.

Blue light and hypoxia/superoxide-induced Fenton reaction have also been identified as contributing factors to ferroptosis of RPE cells. Yang et al. suggested that the exposure of N-retinylidene-N-retinylethanolamine (A2E), a primary element of RPE lipofuscin, to blue light fostered ferroptosis by elevating Fe^2+^ levels and hindering the SLC7A11/GSH/GPX4 axis (87). Ji and colleagues have demonstrated that the specific inhibition of HMOX1 expression significantly suppresses RPE ferroptosis, suggesting that HMOX1 may be a viable target for protecting RPE cells against blue light-induced ferroptosis (88). Henning et al. administered NaIO_3_ with dimethyloxalylglycine (DMOG) to ARPE-19 cells, a treatment that resulted in the stabilization of hypoxia-inducible factors (HIFs). The treatment was found to trigger SOD activity, which in turn drove hydrogen peroxide (H_2_O_2_) production and fostered the Fenton reaction, thereby aggravating oxidative stress-induced ferroptosis by NaIO_3_ (81). Additionally, the depletion of MnSOD (superoxide dismutase) in RPE cells triggered the Hsp70-linked ubiquitin-dependent cleavage of GPX4, exacerbating ferroptosis of these cells (89).

Notably, crosstalk between other forms of PCD and ferroptosis has been indicated by certain studies during RPE cell death. It is shown that necroptosis and ferroptosis of RPE cells share overlapping features, encompassing ROS accumulation and receptor-interacting protein kinase (RIPK) 1/RIPK3 activation, with Necrostatin-1s (an inhibitor of necroptosis) alleviating RPE deterioration in GPX4-deficient mice (84, 90). Additionally, disturbances in autophagy-lysosome degradation process, where the assembly of ATPases was interrupted and the lysosome in RPE cells was alkalinized, led to the digestion of ACSL4 protein, and ultimately to ferroptosis (91).

### 3.2 RGCs

Within the retina, RGCs constitute the exclusive motor neurons capable of projecting their axons into the CNS, the selective and progressive loss of which causes irreversible damage to visual function (92). Though evidence has identified apoptosis as the predominant mode of RGC death in models of several optic neuropathies, strategies targeting it have proved unsatisfactory, with research suggesting that RGCs rescued by Caspase inhibition only achieved partial neuroprotection (93). The distinctive structure of RGCs leaves it susceptible to ferroptosis. The axons of RGCs form long-extending nerve fiber bundles, the structural and functional maintenance of which necessitates the generation of considerable amounts of ROS by mitochondria (94).

Pathologically high intraocular pressure (ph-IOP) is the predominant risk factor for RGC death. An increased Fe^3+^ concentration in peripheral serum was identified in patients with ph-IOP, and abnormal Fe²^+^ accumulation in the retina was observed at 1–8 hours post-ph-IOP (95). The knockdown of NCOA4 increased the level of FTH1 and diminished Fe^2+^ concentrations within the retinas of mice subjected to ph-IOP, indicating ferritinophagy as a primary factor contributing to RGC demise (95). Utilizing mouse models with chronic ocular hypertension, Guo et al. observed the repressed expression of GPX4 in the mitochondria of RGCs, concomitant with elevated levels of mt-ROS (62). It is also conceivable that ph-IOP exerts a detrimental effect on RGCs indirectly through the triggering of RIR injury. In murine models of RIR injury, the overexpression of p53 (a controller in ferroptosis processes) suppressed SLC7A11 expression in conjunction with increased ALOX12 expression, triggering RIR-induced ferroptosis of RGCs (96). It is also reported that the signaling cascades regulating Fe^2+^ production were activated in RGCs isolated 24 h post RIR, which prompted the simultaneous induction of multiple genes implicated in ferroptosis and other types of RCD (97).

It has also been hypothesized that ferroptosis may occur in response to glutamate excitotoxicity, a cellular and molecular process that contributes to the injury of RGCs (98). Intravitreal injection of N-methyl-d-aspartate (NMDA) has been shown to upregulate ACSL3 and Prnp, which are connected with ferroptosis, as well as the accumulation of Fe^2+^ in the retina and RGCs, respectively (99, 100). The m6A RNA modification of HMGCS1 mediated by Mettl3, the inhibition of which alleviated ferroptosis of R28 cells and restored the visual function in rats, was viewed as a key regulator of RGC ferroptosis (101). *In vitro* experiments demonstrated that glutamate induced the enhanced levels of FTL, GPX4, and SLC7A11 in R28 cells, which were reversed through the administration of the inhibitor of p38 mitogen-activated protein kinase (MAPK) pathway (102).

Intriguingly, a recently published article explored the involvement of the gut-retina axis in retinal ferroptosis. Increased levels of indoleacrylic acid (IDA) in the gut microbiota were reported to contribute to its accumulation in the retina, ultimately provoking ferroptosis of RGCs via the AhR/ALDH1A3/FSP1 axis (103).

### 3.3 PR cells

The light-sensitive PR cells in the retina, comprised of cones and rods, are the primary sensory neurons responsible for visual perception. Morphologically, a PR cell’s outer segment is connected to the inner segment through a cilium, with its synaptic ending residing in the outer plexiform layer (OPL) and its soma positioned in the outer nuclear layer (ONL). The loss of the inner and outer segments, as well as the thinning of the ONL, typically contributed to the compromise of visual function (104). Previous studies on PR preservation have emphasized on the suppression of apoptosis/autophagy, the incomplete therapy of which suggested the involvement of other processes in PR death.

Both the outer and inner segments of PR render it exceptionally susceptible to LPO and ferroptosis. The outer segment membranes of PR cells contain an abnormally high concentration of long-chain PUFAs, particularly docosahexaenoic acid (DHA), which account for over half of the total acyl groups (82). In addition, PRs are recognized as the most highly metabolically active cells in the retina, owing to the vigorous oxidative metabolism within the mitochondria exclusively localized in the inner segments of PR cells (105). In wild-type mice given intravitreal iron injections, *in vivo* imaging and quantitative PCR revealed that merely Fe^2+^ induced PR autofluorescence and a raised level of ferroptosis markers, contributing to PR death (106). Building on previous work, a report found that intravitreal injection of FAC caused green AF in the mitochondria-rich PR ellipsoid and then gold AF in the rod OS, where the gold signature is consistent with increased bisretinoid all-trans-retinal (atRAL) (83).

The accumulation of atRAL in PR cells, resulting from the disruption of the retinoid cycle, is tightly aligned with the eventual demise of these cells. A team of researchers has indicated that the initiation of ferroptosis by atRAL is a pivotal factor in the occurrence of PR loss. In the Abca4Rdh8 mouse model, which exhibits defects in atRAL clearance, Chen et al. has identified mitochondrial disruption and system Xc- inhibition in PR cells following exposure to light (107). Considering that heme oxygenase-1 (HO-1) gene KO and treatment with zinc protoporphyrin IX (a HO-1 inhibitor) reduced Fe²^+^ levels and ROS-mediated LPO in PR cells, it has been proposed that HO-1 is a critical modulator of PR ferroptosis induced by atRAL (108). In PR cells loaded with atRAL, c-Jun was phosphorylated by activated c-Jun N-terminal kinase (JNK), which promoted ferritinophagy facilitated by the upregulation of NCOA4, consequently releasing large amounts of labile iron (109). Furthermore, the elimination of GSDME has been demonstrated to repress atRAL-induced PR ferroptosis and retinal atrophy, which is attributed to its capacity to inactivate mt-ROS-induced oxidative stress in Abca4Rdh8 mice (110).

Studies have highlighted several other key regulators of ferroptosis in PR, namely GPX4 and SLC7A11. The unsplicing of GPX4b mRNA, engendered by the suppression of splicing factor prpf8, has been evidenced to diminish GSH and instigate ferroptosis and mitophagy of PR (111). By applying transcriptome sequencing to determine the possible pharmaceutical target of N-methyl-N-nitrosourea (MNU)-induced mouse models, researchers ascertained that ubiquitination and degradation of GPX4, catalyzed by Cullin-7, resulted in PR ferroptosis (112). In the 661W cells treated with H_2_O_2_, there occurred a decline in SLC7A11 and GPX4, the overexpression of which attenuated the production of ROS in PR cells. Additionally, a study that performed bioinformatics and biochemical analyses verified this by showing that knockdown of SLC7A11 or treatment with sulfasalazine (an SLC7A11 inhibitor) exacerbated H_2_O_2_-induced ferroptosis in 661W cells (113).

### 3.4 CECs

The corneal epithelium, consisting of stratified squamous epithelial cells, forms the outermost layer of the cornea as a barrier and maintains the ocular immune homeostasis. Diverse injuries to CECs, which are vulnerable to extrinsic insults (e.g., chemicals, injuries, and light), could potentially compromise the structural integrity and physiological function of the cornea (114).

Evidence has emerged to indicate a correlation between ferroptosis and the risk factors for CECs damage. In human CECs (HCECs) exposed to phenylarsine oxide (PAO), a highly toxic trivalent arsenical, significant oxidative stress was reported by Kandhari et al., accompanied by ferroptotic cell death (115). Okoyeocha and his colleagues investigated the toxic pathophysiological impacts of phosgene oxime (CX), a potential chemical threat agent, in mouse ocular tissue (116). It was demonstrated that exposure to CX resulted in a substantial deterioration of the corneal epithelial layer, concomitant with the upregulation of pathways associated with inflammation and ferroptosis (116).

Cigarette smoke (CS) is another potential risk factor for the ferroptosis of CECs. The exposure to CS triggered CECs ferroptosis, chiefly by increasing intracellular iron levels and fostering LPO, a process that is facilitated by elevated TFRC and ACSL4 expression, respectively (117). Otsu et al. undertook a study to ascertain the molecular mechanism that leads to CEC injury induced by CS extract (CSE). The cleaved form of ferritin was observed in CECs that had been incubated with CSE, a process that induced both LPO and Fe^2+^ accumulation within autolysosomal compartments.

Certain investigations have indicated that multiple CECs wounds are indicative of ferroptosis. It was established that the absence of one GPX4 allele resulted in a considerable delayed recovery of corneal epithelial wounds in GPX4 (+/-) mice, whose corneas had been subjected to n-heptanol (118). In denervated mice that underwent corneal scraping, the delayed wound healing of CECs, potentially induced by TRPV1+ sensory denervation, was alleviated by Fer-1 (119). Another researcher established a delayed CEC healing model by removing the central corneal epithelium of mice that had been treated with hyperosmotic stress (HS) for a week, and found a dysregulation of the ferroptosis pathway and an aggregation of lipid peroxides in these cells (120). Some specific ferroptosis inhibitors, such as Fer-1-loaded liposomes and UAMC-3203, were also developed to facilitate the epithelialization and transparency of the cornea subsequent to alkali burns (121, 122).

Researchers have documented the interplay between autophagy and ferroptosis in CECs. Lipidomic and transcriptomic analyses revealed that lipophagy was activated by Atg5 in HCECs exposed to HS, driving ferroptosis in CECs and thus inducing corneal damage and ocular surface inflammation (123). In HCECs applied with desiccating stress, the CEC ferroptosis was triggered by the aggregation of autophagy impairment-derived sequestosome 1 (SQSTM1), the overexpression of which fostered ferroptotic changes and substantially elevated ACSL4 (124). Additionally, the use of Fer-1 or deferoxamine (DFO) has been shown to activate healthy autophagy flux in hyperosmolarity-induced CECs (125).

## 4 Ferroptosis: a novel therapeutic target

**Figure 4:**
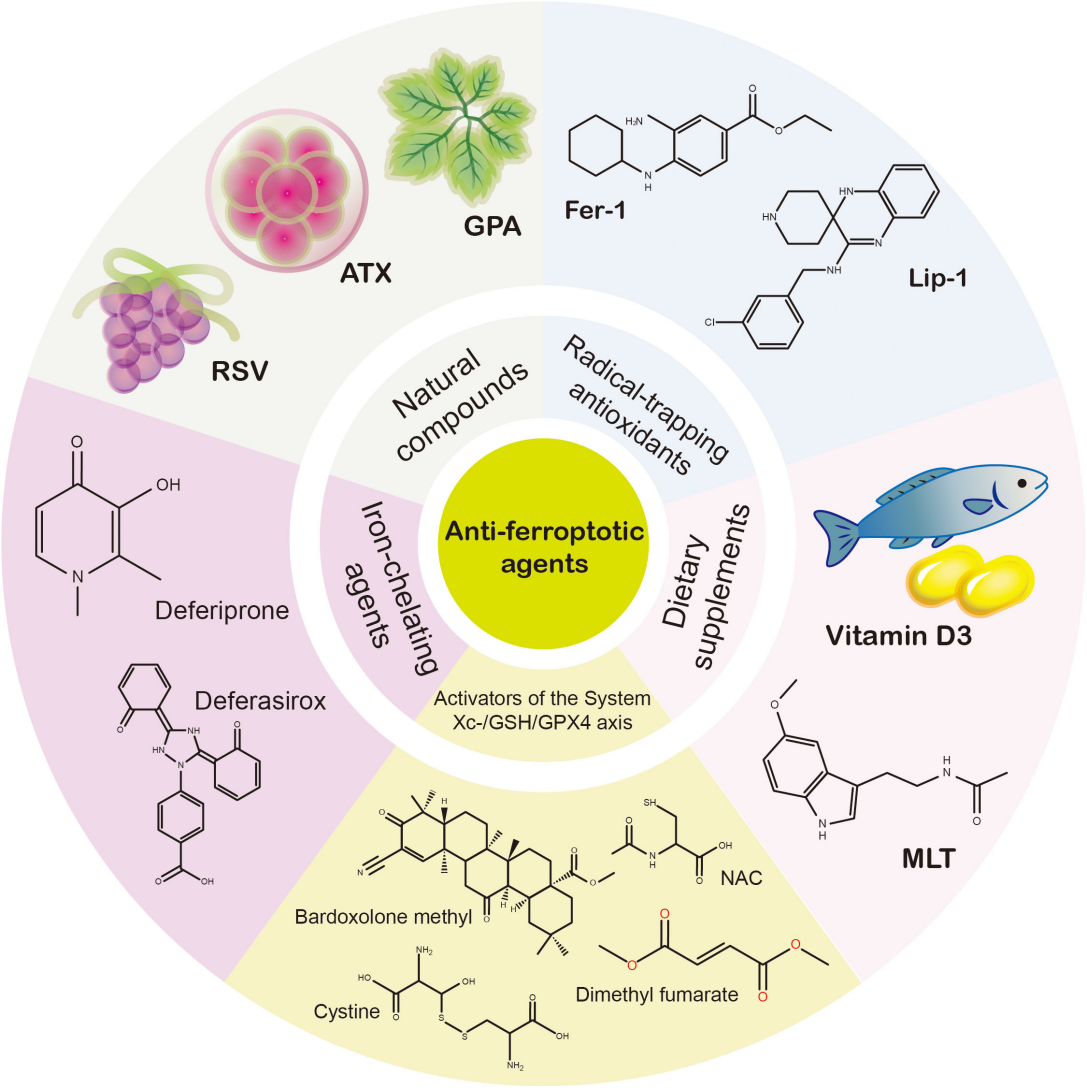
Anti-ferroptotic agents employed in ocular diseases. A plethora of anti-ferroptotic agents employed in ocular diseases have been identified, which primarily consist of radical-trapping antioxidants, iron-chelating agents, activators of the System Xc-/GSH/GPX4 axis, natural compounds, and dietary supplements. Radical-trapping antioxidants, iron-chelating agents, and activators of the System Xc-/GSH/GPX4 axis are direct inhibitors of ferroptosis, whereas natural compounds and dietary supplements mainly act as indirect regulators. Abbreviations: Fer-1, Ferrostatin-1; Lip-1, Liproxstatin-1; GPA, gypenoside A; ATX, astaxanthin; RSV, resveratrol; MLT, melatonin; NAC, N-acetylcysteine.

It has been evident that during the process of ferroptosis, alterations in pathogenesis profoundly affect the normal workings of retinal cells, retinal architecture, and vascular remodeling. Cumulative evidence suggests that both direct inhibitors of ferroptosis, i.e., radical-trapping antioxidants, iron-chelating agents, and activators of the System Xc-/GSH/GPX4 axis, and indirect regulators, i.e., natural compounds and dietary supplements, show promise as anti-ferroptotic agents in promoting ocular cell survival. In addition, therapeutic induction of ferroptosis, which results in the eradication of tumor cells, proves efficient in the management of multiple ocular proliferative diseases (**Figure 4**).

### 4.1 Radical-trapping antioxidants

As one of the first synthetic radical-trapping antioxidants described to block ferroptosis, Fer-1 is widely employed as a reference compound in ocular diseases. Qin et al. evaluated the effects of inhibitors of necroptosis, apoptosis, and ferroptosis (Necrostatin-1, z-VAD-FMK, and Fer-1, accordingly) on primary cultured RGCs suffering RIR injury, with Fer-1 conferring the best efficacy (9). In experimental models of DR, treatment with Erastin and Fer-1 resulted in enhanced antioxidant capacity within the system Xc-, thereby counteracting the effects of Erastin on ferroptosis-induced tissue and cell damage (126). Ferroptosis and deposition of extracellular matrix in HTMCs, the hallmarks of POAG, could be induced by dexamethasone, while the administration of Fer-1 alleviated these effects (127).

Zhang et al. have designed ROS-scavenging and iron-chelating polymers with thioester and thioketal bonds to deliver Liproxstatin-1 (Lip-1), another radical-trapping antioxidant that inhibits ferroptosis, forming a triple therapeutic strategy for acute glaucoma. The oxidation of thioester bonds and the cleavage of thioether bonds in response to cellular uptake by RGCs resulted in the simultaneous release of Lip-1, which effectively inhibited the RGCs’ ferroptosis and preserved visual function (128). *In vivo*, the administration of Lip-1 enhanced the viability and ferroptotic resistance of retinoblastoma cells, which, in turn, exacerbated the tumorigenesis and invasion of retinoblastoma in murine xenograft models (129).

### 4.2 Iron-chelating agents

The utilization of diverse iron-chelating agents in the management of ocular diseases has been referenced, albeit in a limited capacity. For patients diagnosed with acute PACG, oral administration of deferiprone has been observed to traverse the BRB and chelate abnormally elevated Fe^2+^ in the retina following ph-IOP, which contributed to the prevention of RGC ferroptosis and the preservation of visual acuity (95). Sakamoto and his colleagues histologically evaluated the effects of zinc-DFO and deferasirox on retinal excitotoxicity induced by NMDA, which occurs secondary to glaucoma or retinal central artery occlusion. The data indicated that these two iron-chelating agents substantially decreased the intensity of transferrin immunofluorescence, as well as the number of TUNEL-positive cells observed 24 hours subsequent to the administration of NMDA. Network pharmacology and transcriptomic profiling revealed that α-lipoic acid, a naturally occurring iron-chelating agent, exerts a protective effect on AMD via the restoration of the structural integrity and functional capabilities of the retina (130).

### 4.3 Activators of the system Xc-/GSH/GPX4 axis

As a central antioxidant defense pathway in eukaryotic cells, the System Xc-/GSH/GPX4 axis performs a fundamental role in maintaining cellular redox homeostasis and averting ferroptosis (131). Pharmacological activation of the System Xc-/GSH/GPX4 axis has emerged as a promising avenue for the management of ferroptosis-related ocular diseases, as it can suppress ferroptosis by replenishing GSH precursors, upregulating the expression or activity of SLC7A11, or directly stabilizing GPX4 protein to resist its inactivation (132).

As a cysteine prodrug, N-acetylcysteine (NAC) is capable of rapidly replenishing intracellular cysteine pools for GSH biosynthesis, which also enhances the activity of GPX4 (133). Kandhari et al. established an *in vitro* rabbit corneal tissue model of PAO exposure (5 or 10 μg) over a duration of 3, 5, and 10 minutes, followed by treatment with NAC. The study demonstrated that NAC effectively reversed PAO-induced corneal injuries, characterized by a concentration- and time-dependent reduction in epithelial layer thickness, which was mediated by ferroptosis induction (115). Moreover, the administration of NAC was observed to enhance both GSH levels in rats’ eyes and the intracellular GSH pool in RPE cells, predisposing RPE cells to ferroptosis resistance in AMD (134). A retrospective cohort study revealed that NAC users showed a considerably lower risk of developing AMD than non-users, particularly in terms of dry AMD. The author hypothesized that this could be attributed to the anti-ferroptotic property of NAC (135). Intriguingly, Gao et al. revealed that NAC and Fer-1 failed to improve the transcription of retina-specific genes and microphthalmia in 0.5 µM L selenomethionine-induced embryos, in contrast to the ferroptosis activator cisplatin. The study provided indirect evidence that inducing ferroptosis may be a potential therapeutic option for rescuing selenium-induced defects in embryonic eye development (136).

In contrast to the replenishment of GSH precursors, System Xc- activators primarily enhance the expression of SLC7A11 to promote the uptake of cystine, which is both a specific substrate and a direct activator of the system (137). In the study conducted by Ma et al., the viability of cells in Graves’ orbitopathy fibroblasts was compromised by ferroptosis induced by Eastin treatment, which was then reversed by exogenous cystine supplementation (138). Furthermore, the agonists of Nrf2, which serve as pivotal upstream regulators of SLC7A11, function as indirect activators of System Xc-, as they regulate SLC7A11 at the transcriptional level rather than directly modifying the functional activity of the complex (139). As demonstrated by Shimizu et al., dimethyl fumarate, an Nrf2 agonist, exhibited the capacity to protect ARPE 19 cells and mouse retinas from blue light-induced ferroptosis-like injury in AMD, a consequence of promoting Nrf2 nuclear translocation (140). Bardoxolone methyl is a semi-synthetic triterpenoid that is known to activate the Nrf2 signaling pathway. In an experimental model of ischemic optic neuropathy, treatment with bardoxolone methyl exerts a myelin-preserving impact on RGC survival, achieved by enhancing the transcription of ferroptosis-associated protective factors, namely HO-1 and NQO1 (141).

### 4.4 Natural compounds

In parallel, natural compounds may offer alternative options for the treatment of ferroptosis in ocular diseases. A study employing network pharmacology to analyze gypenoside (GP), a main bioactive compound derived from the traditional Chinese herb Gynostemma, highlighted its potential to reduce the effects of crystalline pathomorphological changes of DC by regulating the P53/SLC7A11/GPX4 pathway (142). Chen et al. encapsulated GPA into mPEG-PLGA, producing a spherical nanoparticle (GPA-NP) with a median diameter of approximately 140 nm, which exhibited sustained delivery and enhanced bioavailability of itself. In contrast to GPA monotherapy, GPA-NP was shown to elicit a stronger protective impact against HG-induced ferroptosis in both DR mice and HG-exposed HRMEC by activating the Nrf2/HO-1/GPX4 axis (143).

Astaxanthin (ATX), a carotenoid with inherent antioxidant capabilities, has been documented as a potential mitigator of ferroptosis by acting on the GPX4 pathway in ARC and DED. Extended ATX supplementation, spanning a period of 8 months, led to a partial reversal of Erastin-induced alterations in human LECs from ARC models, which included the suppression of GPX4, an accumulation of ROS, and a decline in mitochondrial membrane potential (144). *In vitro* models of hyperosmolarity-induced DED have indicated that treatment with ATX contributes to an increase of GPX4, promotion of iron storage, and restoration of the mitochondrial structure (125).

In their investigation, Wang et al. explored the potential of resveratrol (RSV) in mitigating ferroptosis in Müller cells during the initial phases of DR. The findings indicated that RSV impeded retinal neurofunctional alterations by augmenting GSH, Nrf2, and GPX4 levels within the retinas of DR rats. In the streptozotocin-induced DR murine model, HG implementation gave rise to a marked decline in the content of GSH, SLC7A11, and GPX4 proteins, which underwent reversal by RSV (145).

The pathogenesis of DR was similarly shown to be improved by 1,8-cineole, a primary ingredient of the volatile oil present in aromatic plants, which considerably restricted the transcription of thioredoxin-interacting protein and ferroptosis in ARPE-19 cells stimulated by HG (146). Zhou and his colleagues isolated a novel polysaccharide, designated THPE2-F, which possesses a mean mass of 480 kDa and is derived from Tetrastigma hemsleyanum Diels et Gilg. Biochemical analyses indicated that THPE2-F has promise as a therapeutic agent for DR, given its capacity to restore GSH levels and to regulate iron levels under hyperglycemic conditions in DR mice (147).

### 4.5 Dietary supplements

Certain fat-soluble vitamins have been demonstrated to possess the capacity to modulate ferroptosis. Chen et al. constructed a BV2 cell OGDR model to investigate the inflammation in microglia under acute glaucoma, where the intervention with vitamin K1 was demonstrated to inhibit microglial ferroptosis and RGCs loss induced by acute IOP elevation (45). In contrast, vitamin K antagonists, exemplified by warfarin, could induce ferroptotic damage within RPE cells, promote the related vascular proliferation, and ultimately, exacerbate CNV lesions, a sequence of events associated with more pronounced and expeditious vision loss in AMD (148). In an HG environment, 100 nM/500 nM 25-hydroxyvitamin D3 has been observed to exert a stimulatory effect on the proliferation of HRMECs, accompanied by an augmentation in GSH, SLC7A11, and GPX4.

Another well-documented dietary supplement in this field is melatonin (MLT), which is mentioned extensively in the context of dry AMD due to its inhibitory effect on ferroptosis in both RPE cells and PR cells. As demonstrated by Wu et al., the probable targets of MLT in amyloid β1-40 (Aβ1-40)-induced AMD are intimately associated with ferroptosis. It was further established that the principal pathways are profoundly associated with the PI3K/AKT/MDM2/P53 axis and that the KO of the MDM2 gene markedly weakens the inhibitory action of MLT (149). Zhi and colleagues carried out an *in vitro* study, exposing 661W cells to NaIO_3_ and later applying varying doses of MLT. The demonstration was made that the alterations in the expression of iron maintenance proteins in PR cells were impeded by MLT, which suppressed the GSK-3β signaling pathway and hindered Fyn-dependent Nrf2 nuclear translocation (150).

## 5 Conclusion

In the review, we have provided a thorough summary of the molecular and cellular mechanisms of ferroptosis, with an emphasis on the interactions between ferroptosis and immune regulation/the ocular microenvironment. Meanwhile, the mechanisms of ferroptosis-related ocular diseases and the therapeutic strategies targeting ferroptosis have also been elaborated. As preclinical studies and clinical investigations have deepened, evidence from molecular, cellular, and histological studies has formed some consensuses regarding the role of ferroptosis in ocular pathophysiology. Ferroptosis is now commonly accepted as a non-redundant contributor to the degeneration of ocular structural cells, with RPE cells and RGCs being the most well-characterized targets. For RPE cells, iron overload-induced LPO and dysfunction of the system Xc-/GSH/GPX4 axis are confirmed as core pathogenic events in AMD (151). Likewise, targeting ferroptosis has become a well-validated therapeutic strategy for ocular diseases. Lip-1 and deferiprone have exhibited consistent neuroprotective effects in preclinical models of glaucoma and DR, while some natural compounds are increasingly recognized for their capacity to modulate the GPX4/Nrf2 axis, thereby suppressing ferroptosis in LECs and Müller cells (75, 125, 126, 128). Lastly, a consensus exists that ferroptosis synergizes with other PCD types in ocular pathologies: autophagy impairment drives ferroptosis of CECs in DED, and necroptosis shares overlapping mechanisms with ferroptosis in RPE cell degeneration (90, 123). This interplay underscores the necessity for combinatorial targeting strategies to attain ideal therapeutic outcomes.

Although advances and consensus regarding ferroptosis in ocular homeostasis and pathogenesis have been achieved, numerous important knowledge gaps and unresolved controversies still exist in this field. For instance, recent studies show that the epigenetic and post-translational mechanisms tightly regulate ferroptosis in other tissues, while their specific roles in ocular cells remain largely unexplored, leaving a critical knowledge gap and hindering the identification of novel regulatory targets (152, 153). As previously discussed, RMECs are highly sensitive to ferroptosis under pathological conditions, as they poorly upregulate GPX4 and SLC7A11, while RPE cells resist ferroptosis via upregulating FTH1 and activating the Nrf2 antioxidant pathway (154). However, the precise molecular differences underlying ferroptosis susceptibility in other retinal cells remain incompletely elucidated, including whether differential expression of regulators such as ACSL4 and HSPB1 accounts for this divergence. One core controversy is the priority of ferroptosis in ocular disease pathogenesis. For example, Tang et al. proposed that RPE degeneration in AMD is predominantly driven by HO-1-mediated oxidative stress rather than ferroptosis. They emphasized that HO-1 functions as an oxidative agonist rather than a driver of ferroptosis, and that ferroptosis occurs only as a secondary event following severe oxidative damage (155). Second, the interaction between ferroptosis and intraocular immunity remains debatable. In autoimmune uveitis, Zhang et al. proposed that TGFBR1 overexpression aggravates retinal inflammation via microglial ferroptosis, yet a contradictory finding indicates no significant association between ferroptosis-related genes and disease progression, suggesting that immune cell infiltration occurs independently of ferroptosis (45, 156). This discrepancy may arise from differences in experimental models and detection methods.

This research field also remains challenged by translational limitations. The first key challenge related to translational limitations pertains to the restriction of ocular drug delivery, primarily attributable to the unique anatomical and physiological barriers of the eye. For AMD, the tight junctions of the BRB could prevent ferrostatin-1or liproxstatin-1, which are hydrophobic small molecules, from efficiently reaching the RPE layer (157). Similarly, deferoxamine, the delivery of which via topical eye drops is limited by poor corneal permeability, fails to achieve effective concentrations in the optic nerve (158). Another critical limitation is the lack of targeted delivery systems, leading to non-specific drug accumulation. The systemic administration of deferasirox often causes off-target effects in the liver and kidneys, while intraocular injection, though improving local concentration, is invasive (159). Additionally, the short intraocular half-life of most ferroptosis modulators, which is less than 1.5 hours in the aqueous humor, necessitates frequent administration, further reducing patient compliance (160). These ocular delivery challenges impede the translation of ferroptosis-targeted therapies, emphasizing the urgency for novel targeted and sustained drug delivery systems.

The clinical translation of ferroptosis-targeted strategies is as well significantly hindered by the restriction of disease models, which involves the discrepancy between preclinical models and clinical settings. *In vitro* models fail to fully recapitulate the complexity of human ocular diseases, leading to inconsistent translational outcomes. For illustration, it is challenging for cell models to replicate the intact BRB, a highly specialized structure composed of RPE cells, RCECs, and tight junctions, which restricts the entry of Fe²^+^ and limits the infiltration of pro-oxidative factors (95). Most cell models rely on high concentrations of inducers (e.g., 10-50 μM Erastin) to trigger acute ferroptosis within 6–24 hours, which cannot simulate the long-term metabolic disorders and heterogeneous pathological features of human DR or AMD (161). As to animal models, the anatomical structure and physiological characteristics of rodent eyes (C57BL/6 mice or Sprague-Dawley rats) differ significantly from those of humans. For instance, mice lack a macula—the key lesion site of AMD where RPE cell ferroptosis is most prominent—which makes it difficult to accurately replicate the ferroptosis-related damage in AMD (107). The discrepancies in the biotoxicity of ferroptosis-inducers in animal models and humans contribute to erroneous assessments of the safety and efficacy of therapies. In human ocular explant models, Erastin triggers adjacent PR damage and disruption of the BRB, which is not observed in rodent models (69).

In recent years, nanocarriers, hydrogels, and targeted carriers for the treatment of ocular diseases by targeting ferroptosis have emerged accordingly. Zhang and co-workers developed a sialic acid-targeting peptide-modified liposome co-loaded with Fer-1 and Cyclosporine A, aiming to achieve long-lasting effects in DED. This multifunctional liposomal formulation exhibits superior aqueous solubility and extended retention time on the ocular surface, further improving its therapeutic efficacy (162). Huang’s research focused on the design of lesion site-targeted MLT-like nanoparticles, named ConA-MelNPs, for the treatment of dry AMD. Intravitreal injection of ConA-MelNPs achieves therapeutic efficacy by impeding RPE cell ferroptosis and lethal oxidative stress, consequently restoring retinal homeostasis (163). Wang et al. established a synergistic therapeutic strategy based on a thermosensitive *in situ* hydrogel consisting of poloxamer and hyaluronic acid, which allows the co-delivery of Fer-1 and antifungal agents. After administration, the hydrogel displays lens-like adhesion to corneal defects and establishes a localized drug depot, which effectively inhibits corneal fibrosis and scar formation of fungal keratitis (164). Gong et al. investigated the therapeutic potential of phytic acid (PA), a natural small molecule with iron-chelating properties, in the form of eye drops and hydrogels for repairing corneal epithelial defects following corneal alkali burns. *In vivo* experimental data demonstrated that PA eye drops contributed to the restoration of corneal structure and function, while hydrogel formulations of PA enabled more sustained drug release and thereby yielded further improved therapeutic efficacy (165). These innovative delivery systems shed new light on the treatment of ferroptosis-associated ocular diseases, offering renewed hope for their clinical management.

Further investigation into ferroptosis in ocular cells, intraocular immunity, and their involvement in ocular pathologies may yield novel mechanistic insights and foster the development of therapeutic interventions. More physiologically relevant models, such as induced pluripotent stem cell-derived RPE (iPSC-RPE) and precision animal models, could help better mimic the progression of AMD and DR, thereby improving the translatability of preclinical findings. In addition, exploring molecular pathways underlying the crosstalk among ferroptosis, ocular immunity, and neuroinflammation may clarify the precise role of microglial activation/Müller cell dysfunction in retinal degeneration. Regarding drug delivery systems, the development of stimuli-responsive and RPE-targeted nanocarriers for intravitreal administration holds great promise to enhance the retinal retention and safety of ferroptosis regulators. Combined, these efforts will drive the translation of ferroptosis-targeted strategies into clinical therapies for blinding ocular diseases.
